# Supporting data for positron emission tomography-based risk modelling using a fixed-instead of a relative thresholding method for total metabolic tumor volume determination

**DOI:** 10.1016/j.dib.2019.104976

**Published:** 2019-12-12

**Authors:** Christine Schmitz, Andreas Hüttmann, Stefan P. Müller, Maher Hanoun, Ronald Boellaard, Marcus Brinkmann, Karl-Heinz Jöckel, Ulrich Dührsen, Jan Rekowski

**Affiliations:** a Department of Hematology, West German Cancer Center, University Hospital Essen, University of Duisburg-Essen, Essen, Germany; b Department of Nuclear Medicine, University Hospital Essen, University of Duisburg-Essen, Essen, Germany; c Department of Radiology and Nuclear Medicine, VU University Medical Center, Amsterdam, the Netherlands; d Center for Clinical Trials, University Hospital Essen, University of Duisburg-Essen, Essen, Germany; e Institute for Medical Informatics, Biometry and Epidemiology, University Hospital Essen, University of Duisburg-Essen, Essen, Germany

**Keywords:** PETAL trial, DLBCL, Positron emission tomography scanning, TMTV determination, Fixed thresholding method, Agreement

## Abstract

Total metabolic tumor volume (TMTV) was measured in 510 patients with DLBCL participating in the PETAL trial. The present data provide information about the prognostic impact of total metabolic tumor volume using the fixed standardized uptake value (SUV_4_) instead of the relative SUV_41max_ thresholding method. A Bland-Altman plot was created to compare both methods. For TMTV assessed by the SUV_4_ method a Cox regression was applied to determine its effect on time to progression, progression-free survival, and overall survival. Kaplan-Meier curves and corresponding hazard ratios were used to estimate the effect of TMTV alone or in combination with interim positron emission tomography response on patients’ survival. The data relate to the research article entitled “Dynamic risk assessment based on positron emission tomography scanning in diffuse large B-cell lymphoma: post-hoc analysis from the PETAL trial” [1].

**Table:** Specifications Table

Subject	Medicine, clinical research
Specific subject area	Hematology
Type of data	Tables Figures
How data were acquired	Clinical assessments, positron emission tomography scanning, TMTV assessment
Data format	Raw and analysed data
Parameters for data collection	Baseline total metabolic tumor volume was measured in a post-hoc analysis including 510 patients with DLBCL participating in the PETAL trial.
Description of data collection	Total metabolic tumor volume was measured using the semiautomatic PETRA accurate tool (v17032017); statistical analyses were performed using SAS software (Version 9.4 for Windows; SAS Institute Inc., Cary, NC) and R (Version 3.6.0 for Windows; R Core Team, 2019)
Data source location	Department of Hematology, Essen, Germany
Data accessibility	Raw data are provided in the supplementary file
Related research article	Christine Schmitz, Andreas Hüttmann, Stefan P. Müller, Maher Hanoun, Ronald Boellaard, Marcus Brinkmann, Karl-Heinz Jöckel, Ulrich Dührsen, and Jan Rekowski Dynamic risk assessment based on positron emission tomography scanning in diffuse large B-cell lymphoma: post-hoc analysis from the PETAL trial European Journal of Cancer

**Table:** 

**Value of the Data** • These data present the prognostic impact for TMTV determination using the SUV4 method. • Our data provide important information on the value of SUV_4_ as a method for TMTV measurement and will be of use for nuclear medicine physicians and haematologists. • Our data may be helpful for further standardization of TMTV assessments that are needed for future studies on risk prognostication and stratification in DLBCL.

## 1 Data

**Fig. 1:**
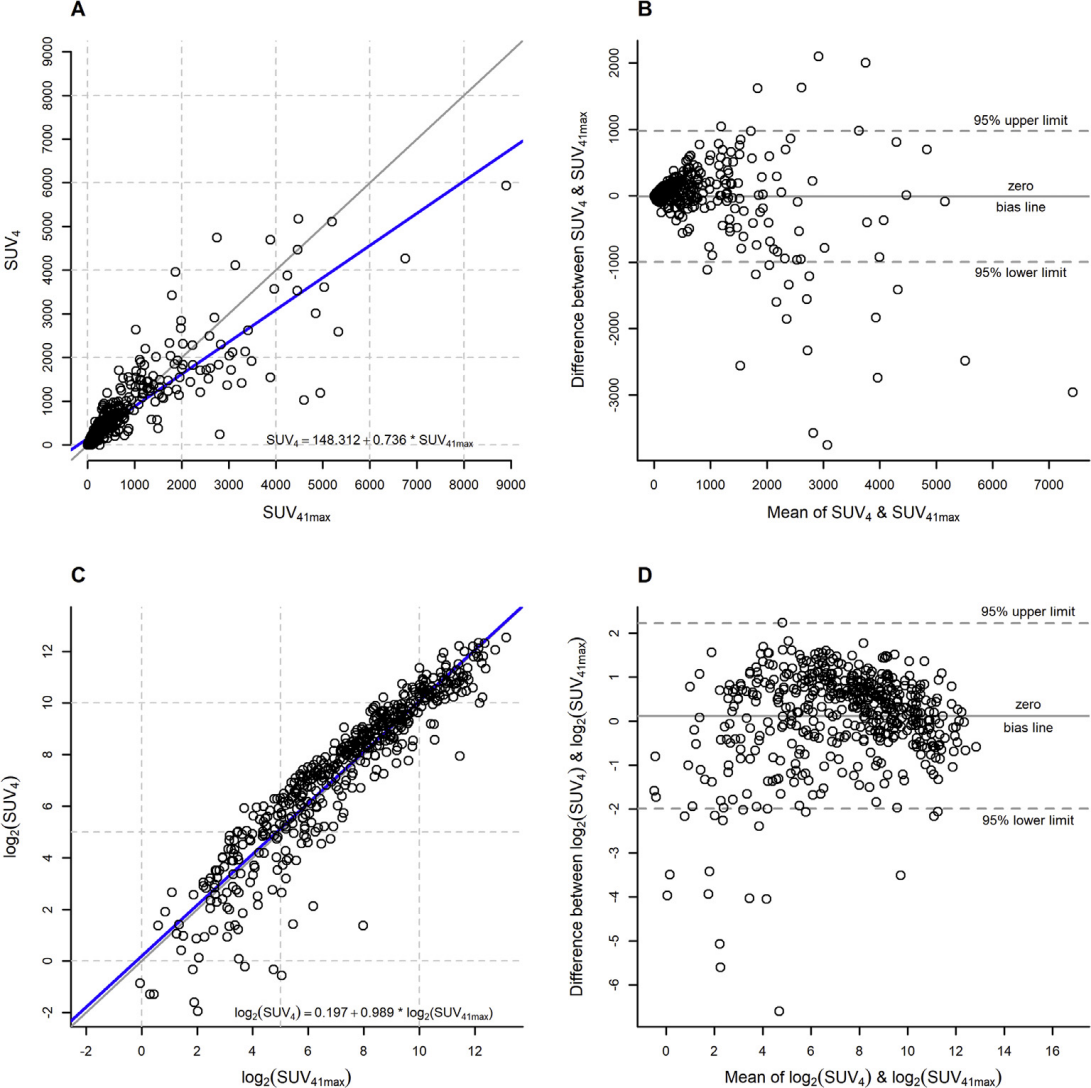
Identity and Bland-Altman plots to assess agreement between fixed and relative threshold methods for TMTV determination.

**Fig. 2:**
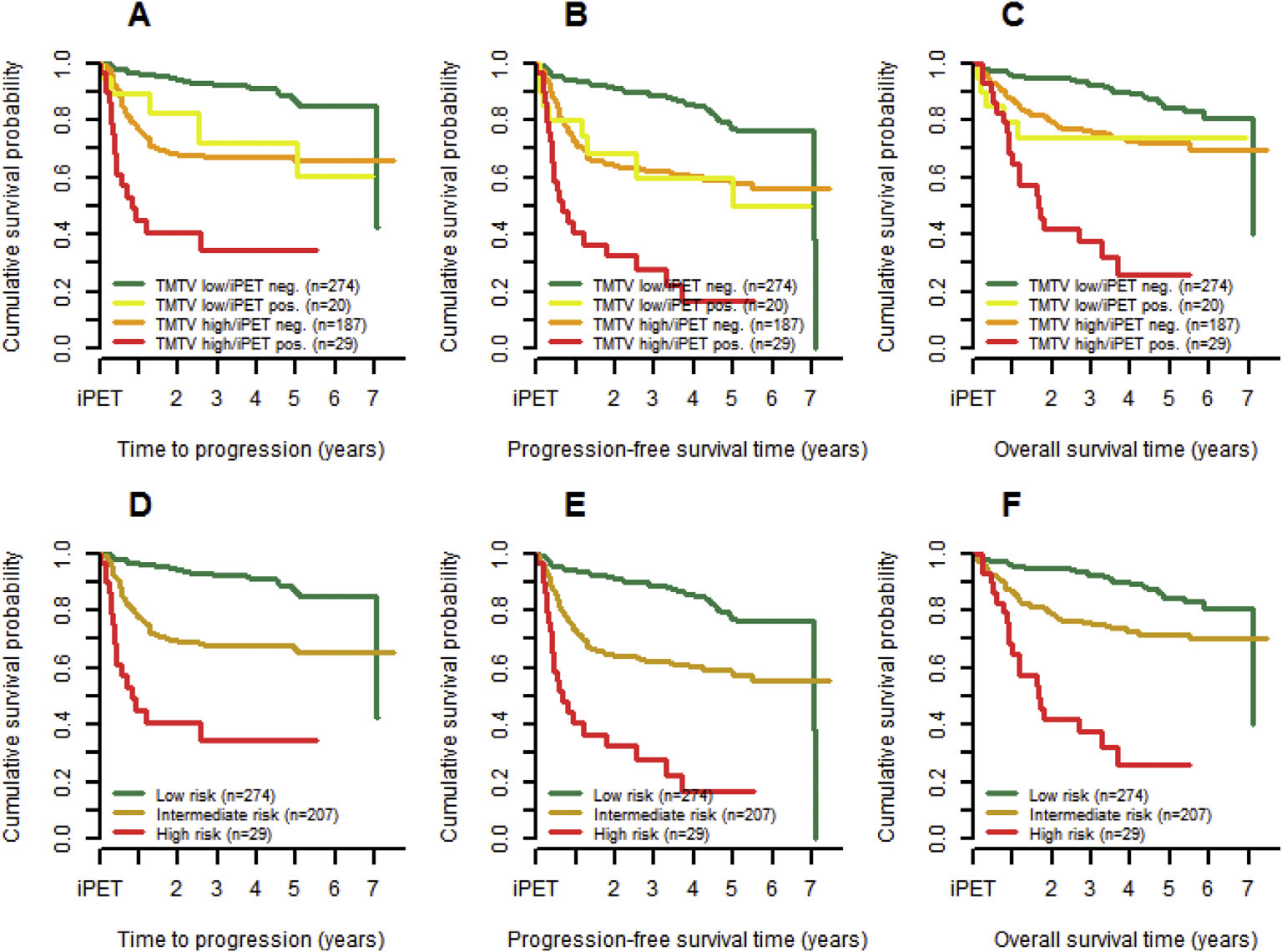
Kaplan-Meier curves for time to progression, progression-free survival, and overall survival in subgroups defined by the dynamic risk model. Panels A–C show the intermediate risk group combinations of TMTV and iPET response separately, while they appear pooled in panels D–F.

We present supporting data belonging to the research article “Dynamic risk assessment based on positron emission tomography scanning in diffuse large B-cell lymphoma: post-hoc analysis from the PETAL trial” [1]. Fig. 1 displays the agreement between the SUV_41max_ and the SUV_4_ method which was assessed using an identity plot (upper left panel) and a Bland-Altman (upper right panel) plot ([2,3]). Lower left and right panels refer to identity and Bland-Altman plots for the log-transformed values of SUV_41max_ and SUV_4_. Cox regression models investigating the effect of interim positron emission tomography-derived (iPET) and International Prognostic Index-derived factors on time to progression (TTP), progression-free survival (PFS), and overall survival (OS) are shown in Table 1. Table 2 presents survival rates and hazard ratios with 95% confidence intervals for time to progression, progression-free survival, and overall survival based on the risk groups of the dynamic prognostic model. Patients with low baseline TMTV according to the SUV_4_ method and good iPET response using the ΔSUVmax method formed a low risk group, while patients with either high TMTV and good iPET response or low TMTV and poor iPET response were defined as an intermediate risk group. Patients with high TMTV and poor iPET response were allocated to the high risk group with corresponding Kaplan-Meier curves within the risk groups of the dynamic prognostic model being displayed in Fig. 2. All raw data are provided in the supplementary file.

**Table 1:** Cox regression modelling of the effect of positron emission tomography-derived and International Prognostic Index-derived factors on time to progression, progression-free survival, and overall survival.

	Time to progression Hazard ratio (95% CI)		Progression-free survival Hazard ratio (95% CI)		Overall survival Hazard ratio (95% CI)	
	Original analysis	Backward elimination	Original analysis	Backward elimination	Original analysis	Backward elimination
Logarithm of baseline TMTV in cm³ (SUV_41max_)	1·19 (1·02–1·40) p = 0·0266	1·21 (1·05–1·41) p = 0·0113	1·14 (1·02–1·29) p = 0·0264	1·20 (1·08–1·33) p = 0·0005	1·24 (1·06–1·44) p = 0·0063	1·36 (1·21–1·53) p < 0·0001
Interim PET response (ΔSUV_max_)	3·51 (2·17–5·66) p < 0·0001	3·47 (2·16–5·58) p < 0·0001	3·31 (2·20–4·98) p < 0·0001	3·35 (2·23–5·03) p < 0·0001	3·44 (2·16–5·49) p < 0·0001	3·57 (2·25–5·68) p < 0·0001
Age >60 years	0·82 (0·55–1·22) p = 0·3367	eliminated	1·42 (1·01–1·99) p = 0·0419	1·46 (1·05–2·02) p = 0·0246	2·22 (1·45–3·38) p = 0·0002	2·31 (1·53–3·49) p < 0·0001
ECOG performance status ≥2	1·16 (0·68–2·00) p = 0·5855	eliminated	1·02 (0·64–1·62) p = 0·9453	eliminated	1·16 (0·69–1·94) p = 0·5763	eliminated
Ann Arbor stage III or IV	2·15 (1·24–3·73) p = 0·0062	1·99 (1·18–3·34) p = 0·0098	1·82 (1·17–2·84) p = 0·0083	1·93 (1·28–2·92) p = 0·0017	1·51 (0·89–2·56) p = 0·1240	eliminated
Elevated LDH	1·79 (1·05–3·07) p = 0·0331	1·73 (1·02–2·94) p = 0·0411	1·41 (0·93–2·13) p = 0·1055	eliminated	1·27 (0·77–2·10) p = 0·3569	eliminated
Extranodal sites >1	0·84 (0·55–1·28) p = 0·4186	eliminated	1·10 (0·76–1·57) p = 0·6237	eliminated	1·00 (0·65–1·54) p = 0·9944	eliminated

**Table 2:** Two-year Kaplan-Meier survival rates for time to progression, progression-free survival, and overall survival within the three groups of the dynamic prognostic model. Hazard ratios between high risk and low risk as well as intermediate and low risk groups for time to progression, progression-free survival, and overall survival with their respective 95% confidence intervals.

	Time to progression	Progression-free survival	Overall survival
2-year survival rate (95% CI)			
Low risk	94·2% (90·6–96·5)	91·4% (87·3–94·2)	95·2% (91·8–97·2)
Intermediate risk	69·2% (62·1–75·2)	64·3% (57·3–70·5)	79·1% (72·8–84·1)
High risk	40·4% (21·8–58·3)	32·5% (16·2–50·0)	41·9% (23·5–59·3)
Hazard ratio (95% CI) High risk vs. low risk	11·20 (6·10–20·58) p < 0·0001	8·51 (5·11–14·16) p < 0·0001	9·03 (5·11–15·96) p < 0·0001
Hazard ratio (95% CI) Intermediate risk vs. low risk	3·56 (2·28–5·56) p < 0·0001	2·68 (1·88–3·81) p < 0·0001	2·39 (1·56–3·66) p < 0·0001

## 2 Experimental design, materials, and methods

The PETAL trial (registered under ClinicalTrials.gov NCT00554164 and under EudraCT 2006-001641-33) is a multicenter randomized controlled study that was approved by the Federal Institute for Drugs and Medical Devices and the ethics committees of all participating sites [4]. Written consent was obtained from all patients. In this trial, all patients aged 18 to 80 with a diagnosis of an aggressive lymphoma were eligible to participate if Eastern Cooperative Oncology Group performance status was ≤3. Patients uniformly received 2 cycles of cyclophosphamide, doxorubicin, vincristine, and prednisone (CHOP) accompanied by administration of rituximab (R) at each cycle in cases of CD20 positivity. An iPET was performed followed by treatment allocation. In case of a favourable iPET response, defined as a reduction of ΔSUVmax by 66%, patients continued therapy with (R-)CHOP for another 4 cycles or 4 cycles of (R-)CHOP with two additional dosages of rituximab. In case of an unfavourable iPET response, patients either received another 6 cycles of (R-)CHOP or switched therapy to receive a more intensive immunochemotherapy that was originally designed to treat Burkitt's lymphoma [6]. Since outcome did not differ within the different treatment arms [4], we were able to combine them for our analyses.

The present analysis is restricted to 510 patients with DLBCL whose baseline PET scans were available for TMTV assessments. Using the semiautomatic PETRA accurate tool [5] TMTV was determined applying the SUV_4_ fixed thresholding method. The software performs a semiautomatic pre-selection of all lesions with an uptake of SUV ≥4. Volumes were then manually adapted, e.g., by removing lesions with physiological uptake. Bone marrow was considered to be involved if there was a focal uptake. Spleen involvement was defined as either focal uptake or a 1.5-fold increased diffuse uptake compared to the liver SUVmean. In contrast, the SUV_41max_ method is a relative thresholding method for TMTV determination. Here, TMTV is obtained by including all volumes whose FDG activity is ≥ 41% of the maximum SUV of each lesion.

Three endpoints were chosen: TTP was defined as time from iPET to disease progression, PFS as time from iPET to disease progression or death from any cause, and OS as time from iPET to death from any cause.

An identity plot of TMTV according to the SUV_41max_ method versus TMTV according to the SUV_4_ method was used to assess the two methods' agreement. Additionally, a Bland-Altman plot investigated this relationship in more detail. Both plots were also produced for the log-transformations of the TMTV variables to facilitate interpretation. As in the companion article, TMTV was combined with iPET response to define a dynamic prognostic model, but here considering TMTV according to the SUV_4_ fixed thresholding method. The best TMTV cut-off to dichotomize patients with respect to SUV_4_ was 345cm³. For iPET response it was 66% according to the ΔSUV_max_ method. The Kaplan-Meier estimator was used to graphically represent TTP, PFS, and OS within the resulting risk groups and to obtain respective 2-year survival rates. Multivariable Cox regression models assessed the prognostic value of SUV_4_ on survival providing hazard ratios with their 95% confidence intervals. While the logarithm of SUV_4_ was considered, the models also included binary variables for iPET response according to the 66% ΔSUVmax criterion as well as for the IPI factors age (>60 years), Eastern Cooperative Oncology Group performance status (≥2), Ann Arbor classification (stage III or IV), lactate dehydrogenase level (>upper limit normal), and extranodal manifestations (>1). The same model was re-run using backward elimination with α = 0.05 as threshold for removing an explanatory variable from the model.
